# Characterization of a Novel Type of HIV-1 Particle Assembly Inhibitor Using a Quantitative Luciferase-Vpr Packaging-Based Assay

**DOI:** 10.1371/journal.pone.0027234

**Published:** 2011-11-03

**Authors:** Gaëlle Gonzalez, Sandrina DaFonseca, Elisabeth Errazuriz, Pascale Coric, Florence Souquet, Serge Turcaud, Pierre Boulanger, Serge Bouaziz, Saw See Hong

**Affiliations:** 1 Université Lyon I & INRA UMR-754, Retrovirus & Comparative Pathology, Lyon, France; 2 Centre Commun d'Imagerie Laënnec, Université Lyon I, Faculté de Medicine, Lyon, France; 3 Laboratoire de Cristallographie et RMN Biologiques, CNRS UMR-8015, UFR des Sciences Pharmaceutiques et Biologiques, Université Paris Descartes, Paris, France; 4 Laboratoire Synthèse et Structure de Molécules d'Intérêt Pharmacologique, CNRS UMR-8638, UFR des Sciences Pharmaceutiques et Biologiques, Université Paris Descartes, Paris, France; National Institutes of Health, United States of America

## Abstract

The HIV-1 auxiliary protein Vpr and Vpr-fusion proteins can be copackaged with Gag precursor (Pr55Gag) into virions or membrane-enveloped virus-like particles (VLP). Taking advantage of this property, we developed a simple and sensitive method to evaluate potential inhibitors of HIV-1 assembly in a living cell system. Two proteins were coexpressed in recombinant baculovirus-infected Sf9 cells, Pr55Gag, which formed the VLP backbone, and luciferase fused to the N-terminus of Vpr (LucVpr). VLP-encapsidated LucVpr retained the enzymatic activity of free luciferase. The levels of luciferase activity present in the pelletable fraction recovered from the culture medium correlated with the amounts of extracellular VLP released by Sf9 cells assayed by conventional immunological methods. Our luciferase-based assay was then applied to the characterization of betulinic acid (BA) derivatives that differed from the leader compound PA-457 (or DSB) by their substituant on carbon-28. The beta-alanine-conjugated and lysine-conjugated DSB could not be evaluated for their antiviral potentials due to their high cytotoxicity, whereas two other compounds with a lesser cytotoxicity, glycine-conjugated and ε-NH-Boc-lysine-conjugated DSB, exerted a dose-dependent negative effect on VLP assembly and budding. A fifth compound with a low cytotoxicity, EP-39 (ethylene diamine-conjugated DSB), showed a novel type of antiviral effect. EP-39 provoked an aberrant assembly of VLP, resulting in nonenveloped, morula-like particles of 100-nm in diameter. Each morula was composed of nanoparticle subunits of 20-nm in diameter, which possibly mimicked transient intermediates of the HIV-1 Gag assembly process. Chemical cross-linking *in situ* suggested that EP-39 favored the formation or/and persistence of Pr55Gag trimers over other oligomeric species. EP-39 showed a novel type of negative effect on HIV-1 assembly, targeting the Pr55Gag oligomerisation. The biological effect of EP-39 underlined the critical role of the nature of the side chain at position 28 of BA derivatives in their anti-HIV-1 activity.

## Introduction

The majority of antivirals against HIV-1 target the following steps of virus-cell interaction : (i) fusion between virus envelope and cell plasma membrane, (ii) reverse transcription of the viral genomic RNA, (iii) provirus integration into the host genome, and (iv) viral protease (PR)-mediated processing of HIV-1 polyprotein precursors (reviewed in [1], [2]). The accumulation of immature, noninfectious virus particles due to unprocessed or partially processed polyprotein precursors could be provoked by anti-PR inhibitors, or by a novel class of antivirals derived from betulinic acid [3]. The prototype of this family of antiviral drugs is the 3-*O*-(3',3'-dimethylsuccinyl)-betulinic acid, abbreviated DSB, known as YK-FH312 [4] or PA-457 [5], and used in the clinics as Bevirimat™ [5]-[10]. In the nanomolar range, PA-457 interferes with the PR-mediated proteolytic cleavage of the Pr55Gag polyprotein substrate at the junction of the capsid (CA) and nucleocapsid (NC) domains, resulting in particles with higher CAp25 content (CAp25 =  CAp24+SP1) and lower infectivity [5], [8], [10], [11]. In the micromolar range and in a heterologous system, we showed that PA-457 has a negative effect on the assembly and egress of virus-like particles (VLP) formed of Pr55Gag [12]. Further confirmation of the structural effects of PA-457 was provided in a recent study, showing that PA-457 used at micromolar concentrations freezes the lattice of protein shell underlying the HIV-1 envelope in its immature configuration [13].

All these studies clearly indicate that the structural component Pr55Gag and its assembly process is a true therapeutic target for anti-HIV drug therapy (reviewed in [14]). An increasing number of inhibitors of Gag assembly are being identified and characterized, e.g. viral core- or cytoskeleton-destabilizing agents (reviewed in [15]), chemicals directed towards the MA, CA or NC domains [16], [17], or Gag-interacting peptides competing with domains of Gag-Gag interaction [18], [19]. As a corollary, there is a need to develop sensitive and reliable bioassays for virus assembly inhibitors, based on simple, easy and low cost experimental approaches mimicking the *in vivo* situation. HIV-1 Gag precursor contains all the morphopoietic information required for the assembly of virus particles, and expression of Pr55Gag in various heterologous cellular [20]–[22], or acellular contexts [23] has proven to be a very convenient method to dissect the natural process which occurs in HIV-1-infected cells. Furthermore, membrane-enveloped HIV-1 VLP produced by recombinant baculovirus-infected insect cells are structurally indistinguishable from immature particles isolated from HIV-1-infected human cells [24], and their high productivity permits valuable statistical analyses of altered morphological and immunological features of Gag mutant particles [24]–[33].

The assay for HIV-1 Gag assembly that we developed in the present study is based on the observation that Vpr and Gag precursor are coencapsidated into immature particles of HIV-1 [34], [35], and on the evidence that Vpr is a *bona fide* structural component of the viral core [36]–[39]. Vpr is copackaged with Gag through the interaction of the N-terminal alpha-helical domain of Vpr encompassing residues 17–33 [40]–[43] with the LXXLFG motif in the p6 domain of Gag [37], [38], [44]–[48]. Taking advantage of this unique property, we fused the luciferase gene from *Photinus pyralis* firefly luciferase to the *vpr* gene of HIV-1, and expressed the luciferase-Vpr fusion protein (LucVpr) in Sf9 cells, using a recombinant baculovirus. When LucVpr was co-expressed with HIV-1 Pr55Gag, luciferase activity was recovered in the extracellular VLP fractions, as a result of the Vpr-mediated encapsidation of the enzyme. The stoichiometry of LucVpr-to-Pr55Gag polyprotein was not significantly different from the Vpr-to-Pr55Gag ratio in HIV-1 virions.

Assembly and egress of VLP could therefore be quantitatively determined in Sf9 cell culture medium using a luciferase-Vpr packaging-based assay, and we used this assay to evaluate and compare the efficacy of a panel of derivatives of betulinic acid (BA) as potential HIV-1 assembly inhibitors. This included the well-documented leader compound PA-457, and five DSB-derived compounds, glycine-conjugated DSB (ST-327), beta-alanine-conjugated DSB (EP-48), free ε-NH2 or ε-N-blocked lysine-conjugated DSB (EP-62 and EP-47, respectively), and ethylene diamine-conjugated DSB (EP-39). We distinguished three types of negative effect on VLP assembly: (i) a progressive, dose-dependent inhibition of the budding and extracellular release of membrane-enveloped VLP at the plasma membrane of Pr55Gag-expressing cells, with no detectable morphological alterations of the VLP compared to control samples, as described in our previous study [12]. This pattern was shown by PA-457, EP-47 and ST-327 (group I compounds). (ii) Group II compounds was exemplified by EP-48 and EP-62, of which the inhibitory effect on VLP production was not specific, as it paralleled a high cytotoxic effect. (iii) In the case of EP-39 (group III), we observed aberrant assembly of Gag polyproteins in the cytoplasm, and their extracellular release as nonenveloped, morula-like Gag particles. The EP-39 activity differed therefore from the antiviral effects previously described for other BA derivatives, *e.g.* the blockage of membrane fusion and entry [49], [50], the inhibition of maturation cleavage CAp25-CAp24 [14], and the inhibition of VLP assembly and budding [12]. The importance of the nature of the side chain at position 28 in the anti-HIV-1 activity of BA derivatives, as evidenced by our experimental data, had important implications in the design of future antivirals belonging to the BA family. Ultrastructural analyses of the atypical Gag particles produced in the presence of EP-39 might also provide indirect clues for a better understanding of the molecular mechanism of the Gag assembly pathway, and the identification of possible Gag assembly intermediate steps.

## Results

### Packaging of Vpr and Vpr-tagged luciferase (LucVpr) proteins into HIV-1 VLP produced in insect cells

**Figure 1:**
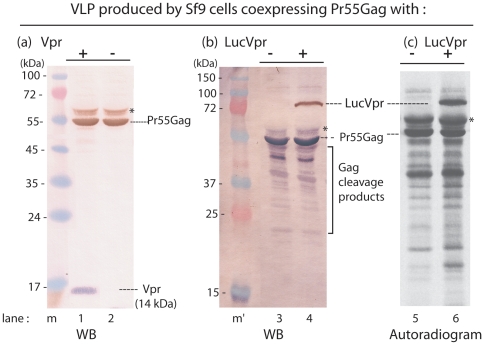
Packaging of Vpr and LucVpr into HIV-1 VLP produced in Sf9 cells. Sf9 cells were infected with (-) AcMNPV-Pr55Gag alone, or (+) coinfected with AcMNPV-Pr55Gag and another recombinant baculovirus expressing (**a**) Vpr or (**b, c** )Luciferase-Vpr fusion protein (LucVpr). Both Vpr and LucVpr were tagged with the His_6_ epitope. VLP were isolated from the culture medium at 48 h pi, using ultracentrifugation in sucrose-D_2_0 density gradient, and each gradient fraction analysed by SDS-PAGE and standard immunoblotting. (**a**), Western blot reacted with anti-Gag polyclonal antibody and peroxidase-labeled anti-rabbit IgG antibody, followed by monoclonal anti-His_6_ tag and phosphatase-labeled anti-mouse IgG antibody. Pr55Gag polyprotein is revealed in brown, Vpr protein (14 kDa) in blue (lanes 1, 2). (**b**), Western blot reacted with anti-Gag polyclonal antibody and phosphatase-labeled anti-rabbit IgG antibody, followed by monoclonal anti-His_6_ tag and peroxidase-labeled anti-mouse IgG antibody. Pr55Gag polyprotein is in blue, LucVpr protein (72 kDa) is in brown (lanes 3, 4). (**c**), Autoradiogram of dried SDS-gel of ^35^S-labeled VLP released from control AcMNPV-Pr55Gag-infected cell cultures (lane 5), or from AcMNPV-Pr55Gag+AcMNPV-LucVpr-coinfected cell cultures, both labeled with ^35^S-methionine and ^35^S-cysteine. Lane m, PageRuler™ pretained protein ladder (Fermentas Inc.). Lane m', Dual Color™ molecular markers (BioRad). Molecular masses are indicated in kiloDaltons (kDa). (*), Asterisk indicates the position of the mono-ubiquitinated Gag polyprotein of 62 kDa, detected by its positive reaction with anti-ubiquitin antibody (not shown).

Vpr has been found to be coencapsidated with the HIV-1 Gag precursor (Pr55Gag) in roughly equimolar ratio to Pr55Gag [45], [46], [51], [52], a stoichiometry which was later re-evaluated to a lower ratio of 1 copy of Vpr per 7 Gag molecules [39]. Sf9 cells were coinfected with two recombinant baculoviruses at equal multiplicity (MOI 10 each), one expressing HIV-1 Pr55Gag and the other Vpr or the fusion protein LucVpr. VLP recovered from the cell culture medium at 48 h pi were analyzed by SDS-PAGE and Western blotting using anti-Gag and anti-His tag antibodies. As shown by immunoblot analysis and autoradiogram of VLP proteins, both Vpr and LucVpr proteins were efficiently packaged into VLP (**Fig. 1**). We also observed a luciferase enzymatic activity associated with extracellular VLP recovered from cells coexpressing Pr55Gag and LucVpr, as detailed below. This suggested that (i) the LucVpr fusion protein was competent for packaging into VLP, and (ii) that the luciferase moiety of LucVpr retained its enzymatic activity after fusion with Vpr. Since some luciferase enzyme could be carried over during VLP purification, the next set of experiments were designed to discriminate between VLP-adsorbed and VLP-incorporated material.

### Specificity of LucVpr encapsidation into VLP : Gag-p6 domain-dependence

To determine whether the luciferase activity that was found associated with HIV-1 VLP represented encapsidated LucVpr, and not enzyme contaminants adsorbed onto VLP, we coinfected Sf9 cells with AcMNPV-LucVpr and AcMNPV-GagΔp6, a recombinant baculovirus which expressed a p6-deleted version of HIV-1 Gag precursor [25], [32], [33]. In previous studies, we have shown that the p6 domain is dispensable for VLP budding and egress from recombinant baculovirus-infected insect cells [12], [32], [33], [53], and, interestingly, that VLP constituted of p6-deleted Gag precursor molecules (GagΔp6 of 47 kDa) showed a more regular shape and higher sphericity than VLP constituted of WT Pr55Gag [28]. Sf9 cells coinfected with AcMNPV-LucVpr and AcMNPV-Pr55Gag (full-length, wild-type Gag precursor) served as positive control, and for negative control for VLP production, Sf9 cells were infected with AcMNPV-LucVpr alone.

**Figure 2:**
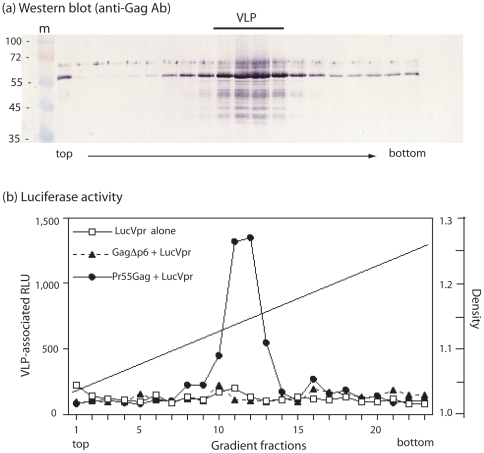
Gag-p6 domain-dependence of LucVpr packaging. Isopycnic ultracentrifugation analysis in sucrose-D_2_0 density gradient of extracellular VLP isolated from Sf9 cell culture medium. **(a)**, Western blot of the gradient fractions analyzed by SDS-PAGE. Blot was reacted with anti-Gag polyclonal antibody and phosphatase-labeled anti-rabbit IgG antibody. Lane m, prestained molecular mass markers (PageRuler™; Fermentas Inc.). The position of VLP (fractions 10–13; 1.12–1.15 in density) is indicated at the top of the panel. (**b**), Luciferase activity was assayed on each gradient fraction, and expressed as relative light units (RLU). Open symbol, Sf9 cells expressing LucVpr alone; filled symbols, Sf9 cells coexpressing LucVpr and full-length Pr55Gag or p6-deleted Gag (GagΔp6). Each gradient fraction was assayed for luciferase activity. Density values are indicated on the right scale.

Cell culture medium of Sf9 cells coexpressing LucVpr and Pr55Gag or GagΔp6 was collected at 48 h pi, using ultracentrifugation through a sucrose cushion followed by a second step of ultracentrifugation in isopycnic gradient [12], [53], [54]. The gradient fractions were analyzed for Gag polyprotein content (**Fig. 2** **a**) and processed for luciferase assay (**Fig. 2** **b**). Samples from cells coexpressing Pr55Gag+LucVpr showed a peak of luciferase activity which coincided with the apparent density of VLP in sucrose-D_2_O density, viz. 1.15–1.25 [12], [53]. In contrast to these control samples, no significant luciferase activity was detected in the VLP-containing fractions from the culture medium of GagΔp6+LucVpr-coexpressing cells : the level of luciferase activity observed was comparable to the background level found in culture medium of cells infected with AcMNPV-LucVpr alone (**Fig. 2** **b**). This result suggested that the luciferase activity that we found associated with membrane-enveloped HIV-1 VLP was packaging-specific, and resulted from a p6-dependent packaging process mediated by the Vpr moiety of the LucVpr fusion protein.

### Quantification of VLP assembly based on luciferase assay, as applied to the prototype assembly inhibitor PA-457

In a previous study we have shown that PA-457 has an inhibitory effect on the assemby and budding of HIV-1 VLP from insect cells expressing the recombinant polyprotein Pr55Gag, with an IC_50_ of 5–6 µg/ml (corresponding to 8–10 µM). We later showed that the sensitivity of VLP assembly to the PA-457 inhibitor did not change in the presence of recombinant Vpr protein coexpressed in Sf9 cells : virus assembly, measured by the level of extracellular VLP, was inhibited at the same dose of PA-457, and with the same IC_50_ value, with or without Vpr [12]. More importantly, we observed that both Pr55Gag and Vpr protein contents of VLP decreased in parallel with the increasing doses of PA-457, suggesting that the level of Vpr encapsidation into VLP reflected well the degree of assembly inhibition [54]. However, it was essential to determine with better accuracy the degree of correlation between the quantities of Pr55Gag and LucVpr proteins coencapsidated into membrane-enveloped, extracellular VLP in the presence of the prototype assembly inhibitor PA-457.

**Figure 3:**
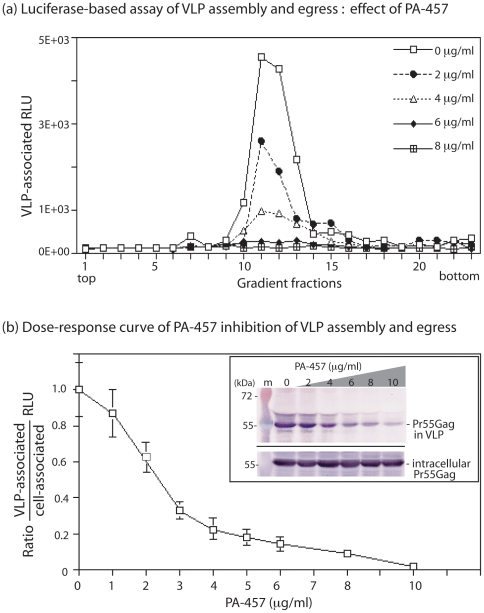
Quantification of VLP assembly and egress using luciferase assay. (**a**), ***Ultracentrifugation analysis of VLP.*** Cells coexpressing Pr55Gag and LucVpr were untreated (control 0) or treated with PA-457 in DMSO for 24 h at 24 h pi, at increasing concentrations as indicated. VLP were isolated from the culture medium at 48 h pi by isopycnic ultracentrifugation in sucrose-D_2_0 density gradient, and assayed for luciferase activity, expressed as relative light units (RLU). (**b)**, ***Dose-response curve of PA-457 inhibitory effect on VLP production***. The ratio of VLP-associated to intracellular luciferase activity was plotted versus PA-457 concentrations. The IC_50_ value obtained was 2.2–2.4 µg/ml. ***Inset*** : VLP production (***top***) and intracellular expression of Pr55Gag (***bottom***) were evaluated in parallel by Western blot analysis using anti-Gag rabbit antibody and phosphatase-labeled conjugate.

Sf9 cells were coinfected with AcMNPV-Pr55Gag and AcMNPV-LucVpr (at a MOI of 10 each), and PA-457 added to the cell culture at 24 h pi at increasing doses, ranging from 0 to 10 µg/ml, and maintained for 24 h. Cell culture medium was then harvested at 48 h pi, subjected to the 2-step ultracentrifugation analysis, and each gradient fraction probed for Gag polyprotein and luciferase activity, as above. We observed that the peak of VLP-associated luciferase activity progressively decreased in a PA-457 dose-dependent manner (**Fig. 3** **a**). The gradient fractions corresponding to the peak of luciferase activity were pooled, and the VLP contained in these fractions were pelleted, lysed and assayed for luciferase content. The luciferase activity was determined in parallel in the corresponding cell lysates, and the values of the ratio of VLP-associated to intracellular luciferase activity were plotted versus the PA-457 concentrations. The curve confirmed the dose-dependent decrease of VLP-associated luciferase in the presence of PA-457 (**Fig. 3** **b**). It correlated with the progressive diminution of the levels of extracellular VLP, as shown by the Pr55Gag signal in Western blot analysis (**Fig. 3** **b inset,** ***top***). The intracellular expression of Pr55Gag remained virtually unchanged within this range of PA-457 concentrations (**Fig. 3** **b inset,** ***bottom***), as already observed [12].

These data suggested that a luciferase assay based on the VLP-packaging of LucVpr fusion protein could legitimately be used to quantitate the VLP production and evaluate the efficacy of antivirals acting at the stage of virus particle assembly and extracellular budding. As exemplified by this particular experiment, the 50% inhibition VLP formation was observed at a PA-457 concentration of 2.2–2.5 µg/ml (**Fig. 3** **b**), corresponding to an IC_50_ of 3.8–4.2 µM. This value was consistent with the IC_50_ value of 8–10 µM previously determined using an immuno-radiochemical assay of VLP [12], a method which could not compare with the luciferase assay, in terms of sensitivity and linearity of the response over a wide range of enzyme concentrations.

**Figure 4:**
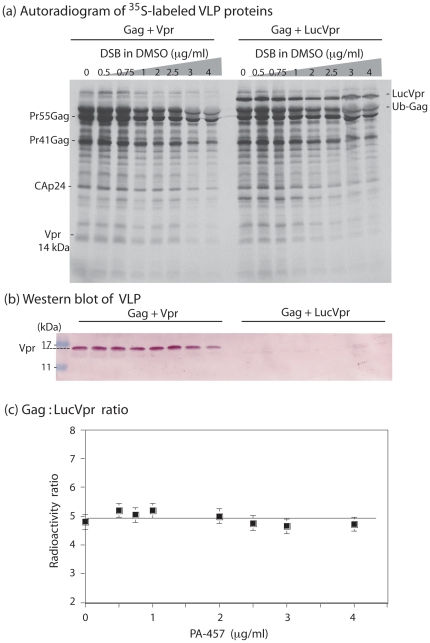
Efficiency of packaging of LucVpr into VLP. (**a**), ***Autoradiogram of ^35^S-labeled VLP*** **.** VLP were isolated from the culture medium of Sf9 cells coinfected with AcMNPV-Pr55Gag and AcMNPV-Vpr (leftmost half of the panel), or AcMNPV-Pr55Gag and AcMNPV-LucVpr (rightmost half of the panel). After purification by ultracentrifugation in sucrose-D_2_0 density gradient, VLP were analyzed by SDS-PAGE, and autoradiography of the dried gel. The position of Pr55Gag and its major cleavage products Pr41Gag, CAp24 and MAp17 are indicated, as well as Vpr, LucVpr and the monobiquitinated form of Pr55Gag (Ub-Gag). (**b**) ***Western blot analysis.*** VLP were analyzed by SDS-PAGE as above, followed by Western blot using anti-Histidine tag antibody and phosphatase-labeled conjugate. Only the portion of the blot showing the Vpr protein of 14 kDa is presented. (**c**) ***Graphic representation of the*** ** ***Gag:LucVpr ratios as a function of the inhibitor concentrations***. Quantification of the VLP content of Pr55Gag, Pr41Gag, CAp24, MAp17, and LucVpr proteins was performed by excision of their corresponding ^35^S-labeled band from SDS-gel as shown in (a), and counting radioactivity in scintillation spectrometer. After correction for the respective number of methionine and cysteine residues in proteins, the values of the Gag:LucVpr ratio were plotted versus the PA-457 concentrations.

However, in the experiments presented in Fig. 3, the possibility that PA-457 (or any member of this class of inhibitors) might negatively interfere with cellular functions indirectly involved in the copackaging of LucVpr and Pr55Gag was envisaged. To address this issue, we labeled baculovirus infected cell cultures with ^35^S-methionine and ^35^S-cysteine, purified the ^35^S-labeled VLP by ultracentrifugation, and analyzed their protein content using SDS-PAGE, and autoradiography and Western blotting (**Fig. 4** **a, b**). Gag and Vpr protein bands were excised from the gel and their respective radioactivity content determined by scintillation counting (**Fig. 4** **c**). Of note, Gag was evaluated as the whole Gag content, including all Gag protein species in our calculation, *i.e.* Pr55Gag, Pr41Gag, CAp24, and MAp17, and corrected for the respective content of sulfur-containing amino acid residues in the different protein species. We found an average molecular ratio of 5.60±0.68 copies of Gag per Vpr molecule (m ± SD; n = 8) in VLP produced by Sf9 cells, a value which was close to the value of 7∶1 reported for HIV-1 virions released by human cells [36]–[39].

The same protocol was applied to VLP isolated from PA-457-treated Sf9 cells coexpressing LucVpr and Pr55Gag. When plotted versus the PA-457 doses, the Gag-to-LucVpr ratios remained virtually constant, regardless of the PA-457 dose (**Fig. 4** **c**), with an average value of 4.92±0.25 copies of Gag per LucVpr molecule (m ± SD; n = 8). The absence of significant decrease of the LucVpr:Gag ratio in the presence of increasing doses of PA-457 therefore excluded a possible direct interference of PA-457 with the LucVpr encapsidation machinery, which could result in apparent lower values of luciferase activity at high PA-457 concentrations.

The minor difference in the mean values of Vpr:Gag and LucVpr:Gag ratios (5.60±0.68 versus 4.92±0.25) was not significant at the *P* = 0.05 level, and suggested that the fusion of luciferase to the N-terminus of Vpr did not significantly alter the encapsidation efficiency of the LucVpr fusion protein, compared to nonfused Vpr. If one considered an average value of 1 copy of Vpr or LucVpr per 5 Gag molecules (ca. 20%) the packaging of Vpr and LucVpr into VLP produced in Sf9 cells was as efficient as the packaging of Vpr into HIV-1 virions (15% Vpr; [39]). This important point validated our method of VLP quantification using Vpr-based luciferase assay and recombinant HIV-1 Gag precursor in the baculovirus-insect cell expression system.

### Evaluation of potential HIV-1 assembly inhibitors using LucVpr packaging-based assay

**Figure 5:**
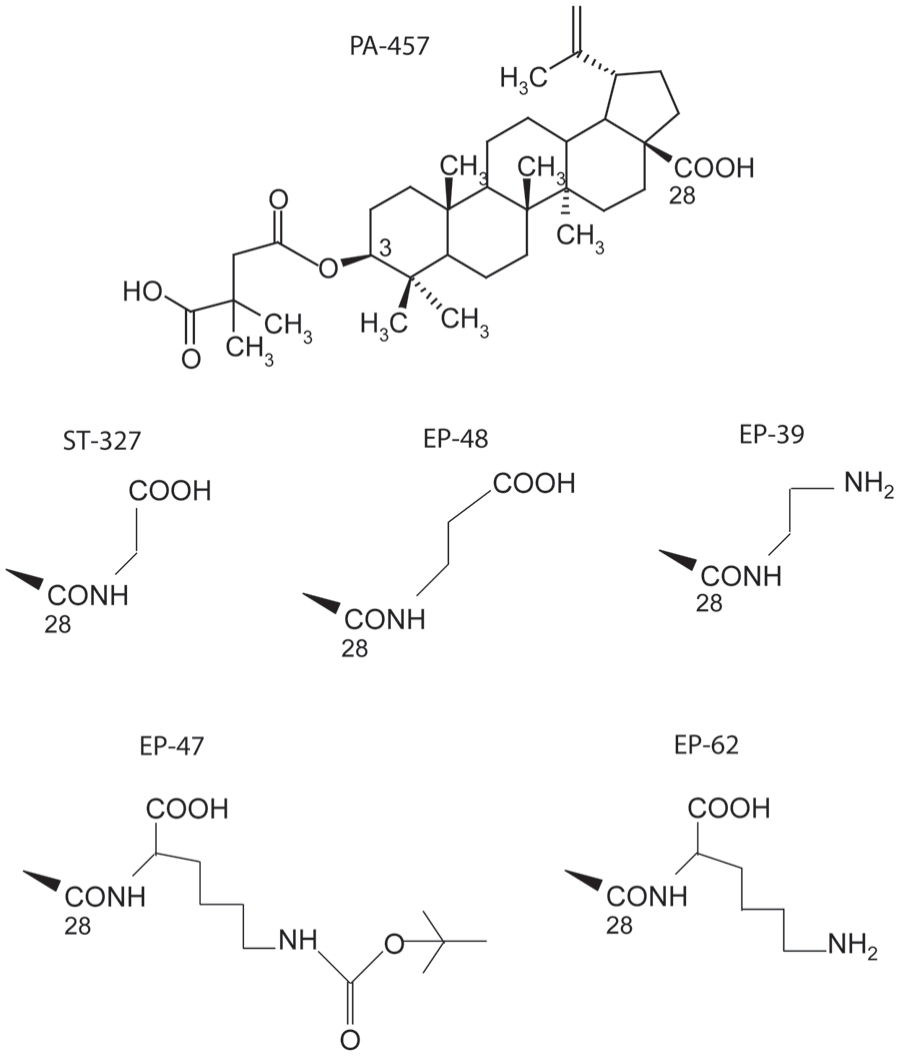
Structure of betulinic acid derivatives. Note that only carbon-3 and carbon-28 are numbered on the PA-457 formula. Compounds ST-327, EP-48, EP-39, EP-47 and EP-62 are schematically represented by their only difference with the leader compound PA-457, i.e. the substituant which amidifies the acidic function carried by carbon-28.

We next evaluated four potential HIV-1 Gag assembly inhibitors derived from PA-457 for their their effect on VLP production. These compounds differed from the leader compound by their substituants on carbon-28 (**Fig. 5**), and might follow a different cellular uptake pathway, and/or distribute differently in the cell compartments, compared to PA-457. These compounds were a glycine-conjugated DSB (ST-327), a beta-alanine-conjugated DSB (EP-48), an ε-NH-Boc-lysine-conjugated DSB (EP-47), a lysine-conjugated DSB (EP-62), and an ethylene diamine-conjugated DSB (EP-39). We compared their effects with those of PA-457 and of the nonsubstituted product, the betulinic acid (BA). BA, PA-457, ST-327, EP-47 and EP-39 were administered to Sf9 cells coinfected with AcMNPV-Pr55Gag and AcMNPV-LucVpr at 24 h pi, and at increasing concentrations. Drug treatment was maintained for a further 24 h, and VLP released in the cell culture medium were pelleted through a sucrose cushion [12]. The amounts of extracellular VLP recovered in the 48 h-pellets obtained in the presence of the different inhibitors were determined using the luciferase assays. After normalization to the luciferase activity determined in the corresponding cell lysates, the values of the ratio of VLP-incorporated to cell-associated luciferase activity were plotted versus the drug concentrations.

**Figure 6:**
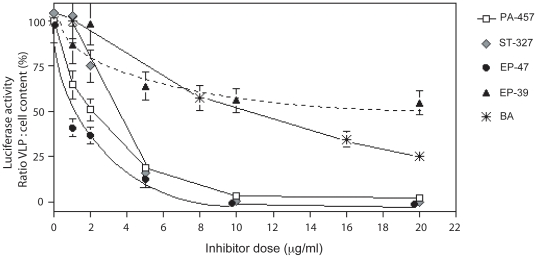
Evaluation of the inhibitory activity of BA, PA-457, ST-327, EP-47 and EP39 on VLP assembly, using luciferase-Vpr packaging-based assay. Aliquots of Sf9 cells coinfected with AcMNPV-Pr55Gag and AcMNPV-LucVpr at equal MOI were treated at 24 h pi with increasing doses of each inhibitor for 24 h. Cells and culture medium were harvested at 48 h pi, and VLP isolated from the culture medium. Cell pellets and VLP were then processed for luciferase assay, and the values of the ratio of VLP-incorporated to cell-associated luciferase activity, in percentage of the control (0 inhibitor), were plotted versus the PA-457 concentrations. In control samples, the fraction of VLP-incorporated luciferase was usually 5 to 7% of the total activity recovered. E.g., in the experiment illustrated here, the activity recovered was 35.5×10^6^ RLU in cell pellets, versus 2.5×10^6^ in extracellular VLP. Note that the inhibition curve of EP-62, which resembled that of ST-327, was not represented for reason of clarity.

Two compounds, EP-48 and EP-62, were found to have a pronounced cytotoxic effect, and no specific effect on VLP assembly (**Table 1**). However, ST-327 and EP-47, as well as the leader compound PA-457, showed a net inhibitory effect on VLP assembly with some differences in their respective efficacy. EP-47 presented the highest inhibitory activity, with IC_50_ ranging between 1 and 2 µg/ml, with a mean value at 1.9 µM, versus 5.1 µM for PA-457 and 5.9 µM for ST-327 (**Fig. 6** and **Table 1**). The selectivity index (SI) values were in the same order of magnitude for EP-47 and PA-457, but 3-fold lower than PA-457 for ST-327 (**Table 1**).

**Table 1:** Efficiency of inhibition of HIV-1 VLP assembly by betulinic acid derivatives (a).

Compound	IC_50_ (µg/ml)	IC_50_ (µM)	CC_50_ (µM)	SI (b)
BA	18.0±3.0	39.4	43.8	1.1
PA-457	3.0±1.0	5.1	93.5	18.7
ST-327	3.8±1.6	5.9	31.1	5.3
EP-47	1.6±0.5	1.9	24.7	13.0
EP-62	3.4±0.4	4.8	3.5	0.7
EP-48	ND (c)	ND (c)	4.2	ND (c)
EP-39	NA (d)	NA (d)	120.0	ND (c)

(a) The mean values (m) for the 50% inhibitory activity (IC_50_) on VLP assembly were given as µg/ml (mean, m ± SEM ; n = 4), or as µM (m). The mean values for cytoxicity (CC_50_) were only given as µM.

(b) The selectivity index (SI) was given by the ratio CC_50_∶IC_50_.

(c) ND, not determined.

(d) NA, not applicable.

Intriguingly, the dose-response curve of VLP assembly inhibition obtained with EP-39 showed a gentle slope until 8 µg/ml, followed by a plateau at 60-50% VLP production at concentrations higher than 10 µg/ml (corresponding to a molarity of 15.6 µM**;** **Fig. 6** and **Table 1**). In addition, EP-39 showed the lowest level of cytotoxicity, compared to the other drugs including BA (**Table 1**). The plateau observed in the inhibition curve of EP-39 implied the occurrence of a residual production of luciferase-positive VLP in the presence of high doses of EP-39. This result was somehow unexpected, since EP-39 inhibited the maturation cleavage of CAp24-SP1 and decreased the virus infectivity with a mean IC_50_ of 16 nM and a SI over 2,000 [55], i.e. a significantly higher efficiency compared to that of the leader compound PA-457. Further analysis of the EP-39 biological effects was then performed to exclude possible faulse negative results.

### Biophysical properties of luciferase-positive particles produced by EP-39-treated cells

**Figure 7:**
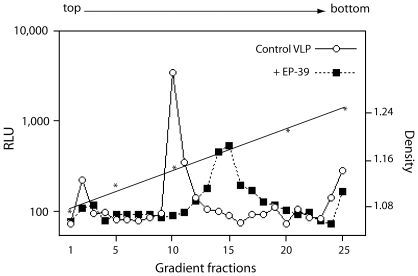
Effect of EP-39 on sedimentation properties of VLP. Cells coexpressing Pr55Gag and LucVpr were untreated (control) or treated with EP-39 (10 mg/ml in DMSO) for 24 h at 24 h pi. VLP were recovered from the culture medium at 48 h pi by ultracentrifugation through a sucrose cushion, and the VLP pellet further analyzed by isopycnic ultracentrifugation in sucrose-D_2_0 density gradient. Gradient fractions were assayed for luciferase activity, expressed as relative light units (RLU). Open symbols, control VLP ; solid symbols, VLP produced in the presence of EP-39.

The extracellular VLP recovered in the 48 h-pellet of culture medium of EP-39-treated cells were analyzed by isopycnic ultracentrifugation in sucrose-D_2_0 density gradient [12], [53], [54], as in the experiments of Fig. 2 and 3. Control VLP released by untreated cells equilibrated at an apparent density of 1.15 (**Fig. 7**), consistent with that of membrane-enveloped retroviral particles [24]. However, residual VLP produced in the presence of EP-39 showed a broader peak of luciferase activity which corresponded to an average density of 1.17 (**Fig. 7**). This suggested an heterogeneity and a change in the composition of the EP-39 VLP compared to control VLP, with a difference in the ratio of protein to lipids consisting of a higher proportion of proteins versus lipids in EP-39 VLP. Our next experiments were aimed at elucidating this point using another approach, based on a structural analysis.

### Structural analysis of EP-39-particles

**Figure 8:**
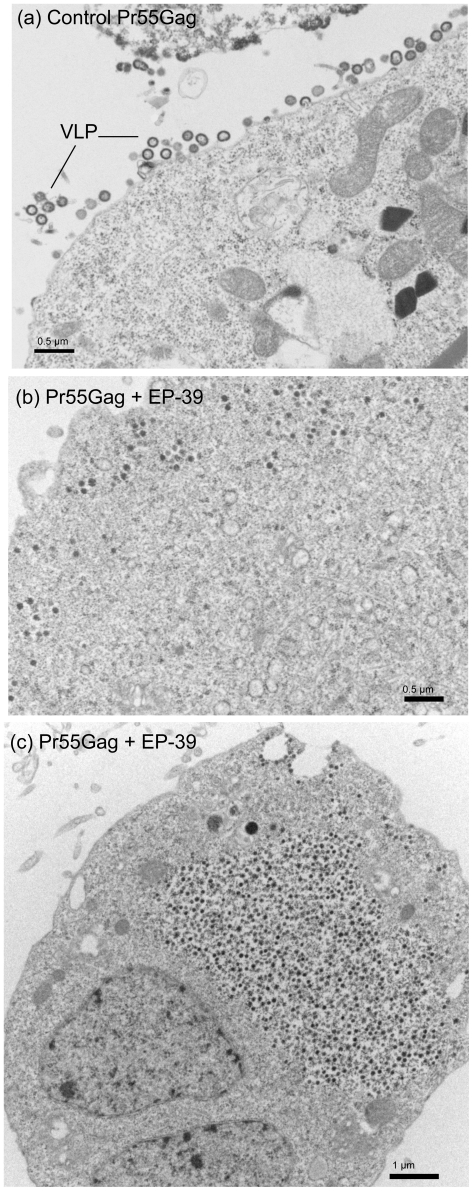
Electron microscopy of Pr55Gag-expressing cells. Samples of Sf9 cells coinfected with AcMNPV-Pr55Gag and AcMNPV-LucVpr at equal MOI, were (**a**) untreated, or (**b, c**) treated at 24 h pi with 10 µg/ml of EP-39 inhibitor for 24 h, harvested at 48 h pi, and processed for observation under the electron microscope.

In a previous study, we have shown that PA-457 added to cell cultures in the micromolar range had a drastic effect on virus assembly. At 10 µg/ml (17 µM), PA-457 totally abolished VLP budding and egress from AcMNPV-Gag-infected Sf9 cells [12]. It also completely blocked the cytoplasmic assembly of core-like particles formed of non-N-myristoylated Gag precursor [12]. By contrast, control, untreated AcMNPV-Gag-infected Sf9 cells were decorated with VLP budding in abundance from the plasma membrane (**Fig. 8** **, a**; and also refer to [12], [28], [30], [32], [33], [53]). To further investigate the molecular and cellular basis for the difference in the inhibitory effect between the three inhibitors PA-457, ST-327 and EP-47 on one hand, and EP-39 on the other hand, we examined by electron microscopy (EM) AcMNPV-Gag-infected Sf9 cells treated with the different drugs at 5 and 10 µg/ml for 24 h at 24 h pi. The electron microscopy (EM) pattern shown by ST-327- and EP-47-treated cells was similar to that of cells treated with PA-457 at the same concentrations (not shown; refer to [12]): a net inhibitory effect on VLP assembly and budding was observed with ST-327- and EP-47.

**Figure 9:**
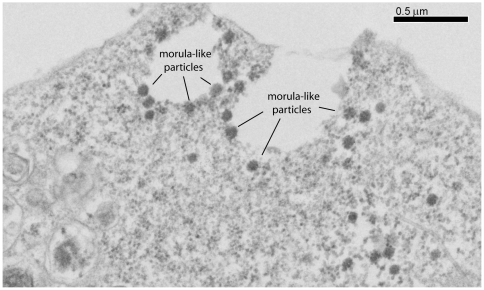
Electron microscopy of EP-39-treated, Pr55Gag-expressing cells. Samples of Sf9 cells coinfected with AcMNPV-Pr55Gag and AcMNPV-LucVpr at equal MOI, were treated at 24 h pi with 10 µg/ml of EP-39 inhibitor for 24 h, harvested at 48 h pi, and processed for observation under the EM. Note the morula-like shape of electron-dense particles of ca. 100 nm in diameter, some of which in the process of egressing into the extracellular milieu.

By contrast to Pr55Gag-expressing Sf9 cells treated with PA-457, ST-327 or EP-47, or to control, untreated cells showing membrane-enveloped VLP in the process of budding out (**Fig. 8** **a**), a different type of cellular response to EP-39 was observed. Electron-dense particles of ca. 100 nm in diameter were observed within the cytoplasm, (i) isolated or grouped as small clusters surrounded by a membrane (**Fig. 8** **b**), or (ii) accumulated in numbers in large cytoplasmic inclusions (**Fig. 8** **c**); (iii) occasionally, 100 nm-particles were seen in the process of egressing into the extracellular milieu, directly or indirectly via vesicles opening to the milieu (**Fig. 9**). These different types of EM patterns often coexisted in the same cells.

**Figure 10:**
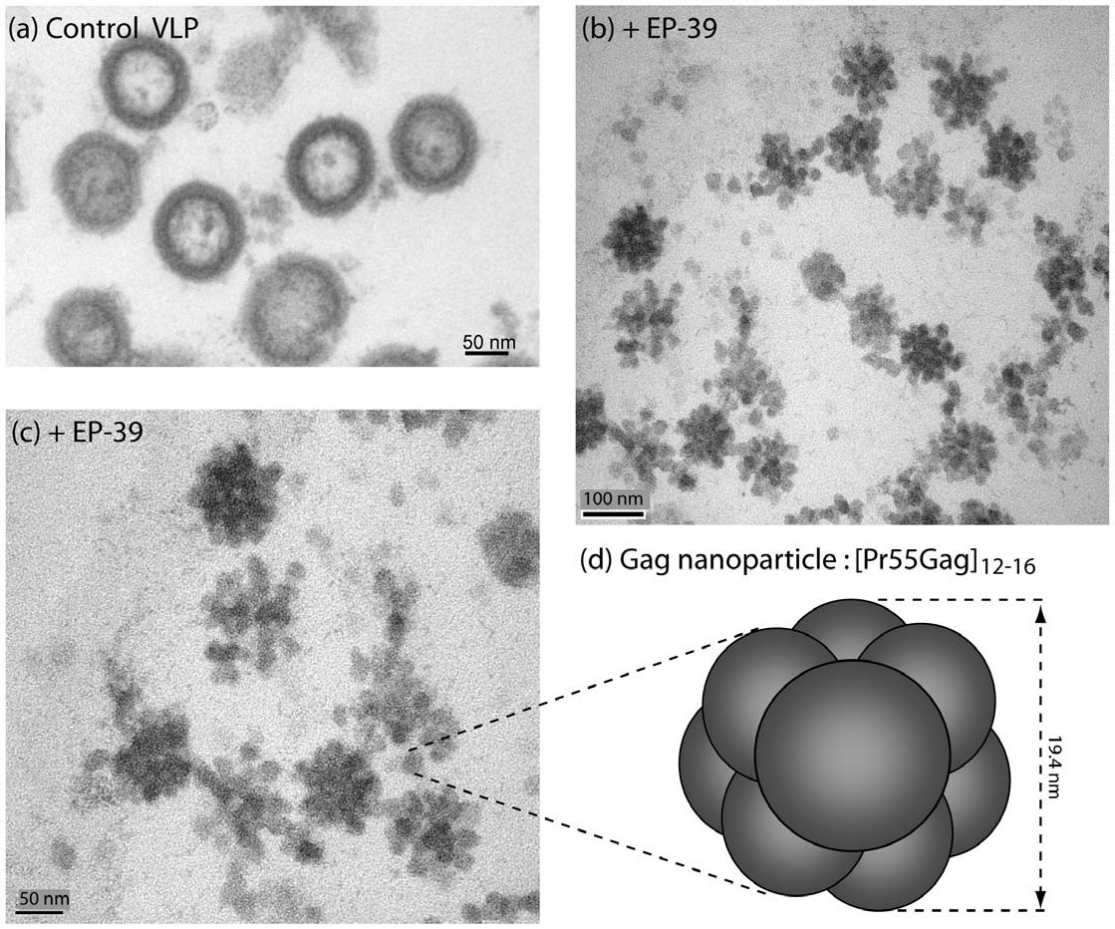
Structural analysis of extracellular EP-39-induced morula-like particles. Sf9 cells infected with AcMNPV-Pr55Gag were untreated (**a**) or treated (**b, c**) at 24 h pi with EP-39 (10 µg/ml) for 24 h. Cell culture medium was harvested at 48 h pi, VLP pelleted by ultracentrifugation, and processed for EM analysis. Electron-dense 100-nm particles are viewed at low (**b**) and high (**c**) magnification, respectively. Control, membrane-enveloped VLP released from untreated cells (**a**) are shown at the same magnification as in (**c**). (**d**), Hypothetical model of an EP-39-induced nanoparticle of ca. 20 nm in diameter, composed of 12 to 16 copies of Pr55Gag assembled with a dodecahedral or octahedral symmetry.

The 100-nm particles assembled in EP-39-treated cells differed structurally from the nonenveloped, intracytoplasmic core-like particles of 100–130 nm in diameter assembled by non-N-myristoylated Pr55Gag [12], [28], [30], [32], [33], [53], and from the extracellular, membrane-enveloped VLP released by N-myristoylated Pr55Gag-expressing cells (**Fig. 10** **,** compare panels **a** and **b**). At high magnification, substructures were discernible, conferring to EP-39-induced 100-nm particles the aspect of morulae (**Fig. 10** **, c**). Morula-like particles had a diameter ranging from 87 to 120 nm (mean diameter, m ± SD = 109.1±9.2, SD, *n* = 14). Each morula appeared to be constituted of bead-shaped subunits (or nanoparticles) ranging from 14 to 27 nm in diameter (m ± SD = 19.4±3.2; *n* = 17). Many morula-like particles were found to be irregular in shape, and/or in the process of dismantling and releasing isolated nanoparticles (**Fig. 10** **c, d**).

**Figure 11:**
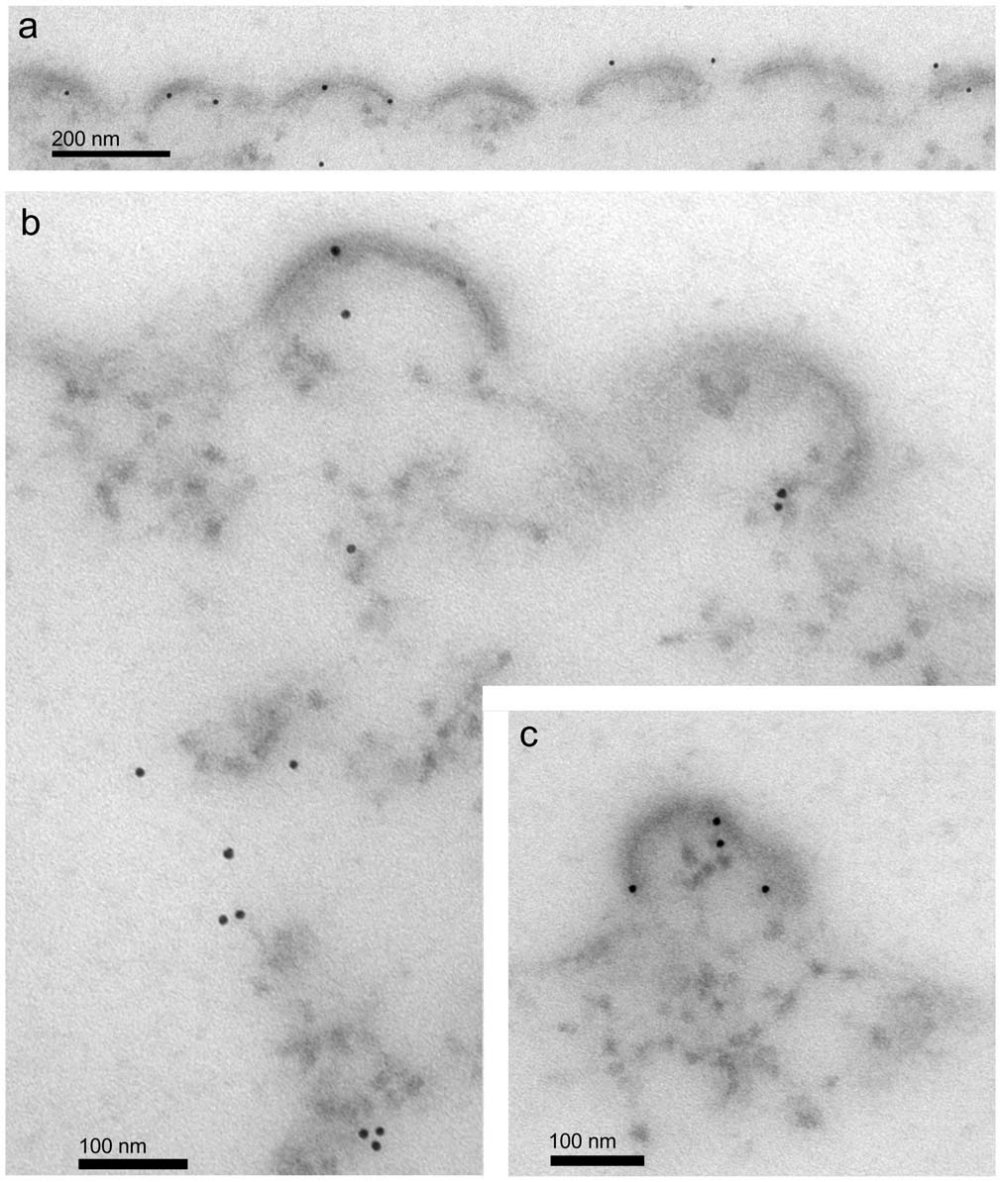
Immunoelectron microscopy (IEM) of EP-39-treated, Pr55Gag-expressing cells. Samples of AcMNPV-Gag-infected Sf9 cells were treated at 24 h pi with EP39 (10 µg/ml) for 24 h, harvested at 48 h pi, fixed and processed for IEM analysis. Ultrathin sections were incubated with rabbit anti-Gag antibody followed by 10-nm colloidal gold-tagged goat anti-rabbit IgG antibody. Panel (**a**) shows a festooned aspect of the cell surface, suggesting an abortive budding of particles. Panels (**b**) and (**c**) show immunogold-labeling associated with cytoplasmic 20-nm nanoparticles and with aberrant particles protruding from the plasma membrane. Note that the fainter staining of the specimens, compared to conventional electron microscopy (refer to Fig. 8-10), was destined to enhance the contrast between the colloidal gold grains and the background.

Immunoelectron microscopy (IEM) of EP-39-treated cells showed colloidal gold grain-tagged anti-Gag antibody localized at the plasma membrane (**Fig. 11** **a**), the cellular compartment where N-myristoylated Gag polyproteins were addressed. The immunogold anti-Gag labeling was found to be associated with protrusions of the plasma membrane with irregular shapes (**Fig. 11** **a-c**), suggesting an abortive or aberrant Gag budding. Intracytoplasmic 20-nm nanoparticles were also labeled with immunogold grains (**Fig. 11** **b, c**). Interestingly, 20-nm nanoparticles were observed in close vicinity of, or even within aberrant VLP (**Fig. 11** **b, c**), a pattern which suggested a continuum of structural elements between nanoparticles and the aberrant larger particles assembled in the presence of EP-39.

### Chemical cross-linking of Pr55Gag in EP-39-treated and untreated cells

**Figure 12:**
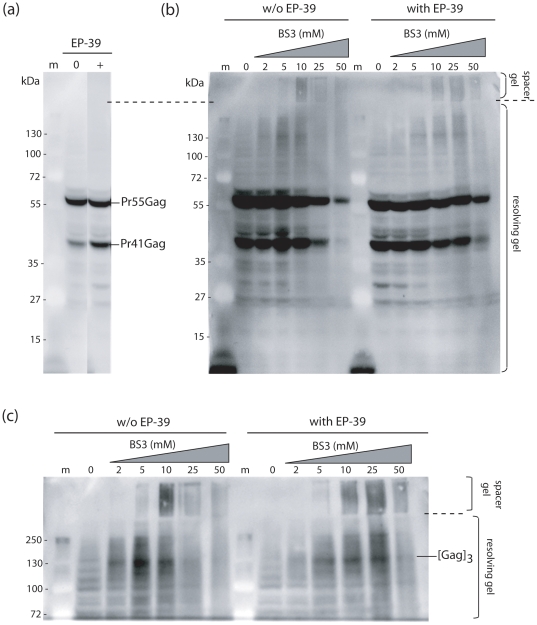
BS3 cross-linking of intracellular Pr55Gag in untreated and EP-39-treated cells. Luminograms of SDS-PAGE and Western blot analysis of recombinant Gag polyproteins cross-linked *in situ* in untreated and EP-39 treated (10 µg/ml) Sf9 cells at 48 h pi. The spacer gel (delineated with dotted lines) was kept intact during the transfer of proteins to the membrane, as it potentially contained high order oligomers of Gag or/and aggregates of high molecular mass, too large to enter the resolving gel. Panel (**a**) corresponds to underexposed control lanes 0 (without BS3 cross-linking) from the blot shown in panel (**b**). Panel (**c**) is an enlargement and overexposure of the top of panel (**b**).

To further analyse the mechanism of the effect of EP-39 on Gag polyprotein assembly, untreated and EP-39-treated (10 µg/ml), Pr55Gag-expressing Sf9 cells were incubated with increasing concentrations of the chemical cross-linker BS3 at 48 h pi for 30 min at room temperature, then lysed in hypotonic medium in the presence of BS3 used in the same range of concentrations. The oligomeric status of the Gag proteins was assessed by SDS-PAGE and Western blot analysis, using anti-Gag antibody, peroxidase-labeled complementary antibody and enhanced chemiluminescence (ECL). In control, non-cross-linked samples, the proportion of Pr41Gag (the major spontaneous cleavage product of Pr55Gag) was higher in EP-39-treated cells compared to untreated cells (**Fig. 12** **a**). This suggested that EP-39 modified the conformation of Pr55Gag and made it more sensitive to cellular proteases. In cross-linked samples without EP-39 treatment, the band of Pr55Gag monomers decreased rapidly at BS3 concentrations higher than 10 mM BS3, and in a dose-dependent manner (**Fig. 12** **b**, leftmost half of the blot, lanes 10, 25, 50). The decrease was less pronounced in EP-39-treated samples (**Fig. 12** **b**, rightmost half of the blot, lanes 10 to 50).

Differences in the pattern of Pr55Gag oligomers between untreated and EP-39-treated cells were also observed, as evidenced on overexposures and enlargements of the luminograms of Western blots (**Fig. 12** **b, c**). A discrete band of anti-Gag reacting protein migrating with an apparent molecular mass of 140–150 kDa and compatible with the status of Gag trimers, was detected at between 2 and 10 mM BS3 in control samples. This band disappeared at higher BS3 concentrations, while, in parallel, anti-Gag reacting material of high molecular mass became visible as a smear within the spacer gel or the loading wells (**Fig. 12** **b** and **c**, leftmost half of the blot, lanes 10 and 25). In EP-39-treated samples however, the band of putative Gag trimers progressively was maximum at 25 mM BS3, and still detectable up to 50 mM (**Fig. 12** **b** and **c**, rightmost half of the blot). This suggested that EP-39 favored the occurrence and/or the stability and persistence of Gag trimers, versus higher order oligomers, as compared to control samples. The cross-linking pattern of Gag *in situ* confirmed our EM observation, and indicated that the Gag oligomerization status and the mode of particle assembly were different in untreated and EP-39-treated cells.

## Discussion

Several methods have been proposed to quantify the HIV-1 assembly and budding process. Conventional techniques such as CAp24 immunoassays used for clinical samples have the inconvenience of not discriminating between soluble CAp24 protein released into the extracellular medium and CAp24 protein incorporated into membrane-enveloped virus particles. On the other hand, virus load assays based on genome copy number determination explore the genome encapsidation process and not specifically the capsid assembly, although RNA encapsidation and virus assembly have been shown to be two linked phenomena [56]–[59]. Several laboratories, including ours, have fused an enzyme to the Gag precursor, e.g bacterial beta-galactosidase [31], [60] or firefly luciferase [22], to quantitate VLP in cell culture supernatants using enzymatic assays. Other methods have preconized the fusion of Gag to enhanced green fluorescent protein from jellyfish *Aequorea* (eGFP), to be able to monitor Gag assembly and budding by flow cytometry, or by fluorescence microscopy *in situ* in cell compartments [61], [62].

Such methods present severe inconveniences and limitations. (i) The addition of a protein tag to the Gag prolyprotein can only be designed at the Gag C-terminus since the N-myristoyl group and the N-terminal stretch of basic residues in the MA domain must be kept intact for plasma membrane addressing and extracellular budding. (ii) Fusion of Gag sequence to an enzyme can be detrimental to the enzymatic activity, in particular for oligomeric enzymes whose activities depend on a specific oligomeric status incompatible with the Gag oligomers occurring during VLP formation [63], [64]. (iii) On the Gag side, the fusion of a protein sequence to the C-terminus of Gag precursor can provoke a steric hindrance which impairs Gag multimerization, assembly and/or budding, or cellular release. This was the case for Gag-β-galactosidase or Gag-protease fusion constructs : the resulting VLP were irregular in shape and size, and hardly mimicked the natural process of virus assembly and egress [28], [31]. Likewise, Gag-GFP fusion protein expressed alone has been found to follow a cellular trafficking pathway different from that of WT Gag in the context of natural HIV-1 cellular infection [61].

Vpr is one of the auxiliary proteins of HIV-1 which is involved in the nuclear import of preintegration complex and cell cycle arrest at the G2 phase of HIV-infected proliferating cells [65]. Vpr is a low-molecular-weight protein (14 kDa) which is a structural component of the infectious virions [46], [51], [52]. Vpr interacts with Gag and is packaged at high copy numbers into immature particles of HIV-1 [39], and this property has been advantageously used to package various Vpr-fused proteins, including antiviral factors [66]–[68]. The β-lactamase-Vpr construct was the first fusion protein used to develop a sensitive Vpr-enzyme based assay to monitor a specific step of the HIV-1 infection cycle, the HIV-1-host cell fusion and entry [69]. In the present study, we took advantage of the Vpr packaging property to construct a recombinant baculovirus expressing a luciferase-Vpr fusion protein (LucVpr), with the luciferase domain at the N-terminus and the Vpr moiety at the C-terminus of the fusion, separated by a flexible linker containing an oligo-histidine tag. When coexpressed in Sf9 cells with the HIV-1 Gag precursor using recombinant baculoviruses, the fusion protein LucVpr was coencapsidated with Gag as efficiently as Vpr alone, with an average ratio of Gag to Vpr molecule of 4.9 for LucVpr, versus 5.6 for Vpr.

The production of HIV-1 VLP by recombinant baculovirus-infected Sf9 cells has been shown to be high, with as much as 10^5^ VLP per cell released in the extracellular medium at 48–72 h pi [33]. It has been reported that one immature HIV-1 particle contains approximately 5,000 copies of closely packed Pr55Gag protein [70], [71]. We estimated the VLP yields by scanning the band of Pr55Gag in Coomassie blue-stained gels, using BSA as protein standard. The Gag signal recovered in the VLP fractions of the linear sucrose-D_2_0 gradient (refer to Fig. 3) corresponded to a total population of 2×10^10^ to 5×10^10^ VLP recovered from 2×10^6^ Sf9 cells. Based on our calculation of the Gag:LucVpr ratio of about 5∶1, the LucVpr content was 1,000 LucVpr molecules per VLP. This implied that the luciferase signal emitted by the whole VLP population corresponded to a total of 2×10^13^ to 5×10^13^ molecules of LucVpr. Since the molecular weight of the fusion protein was 72 kDa (i.e. 6×10^13^ molecules corresponded to 7.2 µg LucVpr protein), the total number of VLP-incorporated LucVpr molecules (2×10^13^ to 5×10^13^) was equivalent to 2.4 to 6.0 µg LucVpr protein in the whole VLP population.

It is generally estimated that purified recombinant luciferase enzyme generates 10×10^3^ to 30×10^3^ RLU per µg luciferase protein. The luciferase signal corresponding to 2.4 to 6.0 µg LucVpr protein would theoretically range from 24×10^3^ to 180×10^3^ RLU. The experimental values of luciferase activity recovered from the sucrose-D_2_0 gradient fractions varied between 5×10^3^ and 30×10^3^ RLU, depending on the experiments (refer to Fig. 3 and Fig. 4 a). The relatively lower recovery in luciferase activity, compared to the theoretical value calculated from the packaging efficiency of LucVpr protein into VLP, could be due to: (i) an incomplete dissociation of certain VLP and the inaccessibility of some Gag-copackaged luciferase to its luciferin substrate; (ii) a lower enzymatic activity of the LucVpr fusion compared to nonfused, natural luciferase enzyme; (iii) the quenching of the luciferase activity due to residual binding of the Vpr domain of LucVpr to the p6 domain of the Pr55Gag precursor; (iv) all three mechanisms.

Despite these minor inconveniences, the luciferase-based quantification of VLP production by insect cells, as described in the present study, represented a simple, rapid and low-cost assay for the screening of potential HIV-1 assembly inhibitors in a living cell system. We then applied this assay to the biological characterization of a panel of betulinic acid derivatives which only differed from the leader compound DSB (or PA-457) by their substituant on carbon-28. The antiviral PA-457 has been identified as an inhibitor of HIV-1 maturation in the nanomolar range [10], and, in the micromolar range, as an inhibitor of VLP assemby [12], [54] and a stabilizer of virus particles in their immature state [13]. We found that the glycine-conjugated DSB (ST-327) and e-N-amidified, Boc-lysine-conjugated DSB (EP-47) exerted a similar type of viral assembly inhibition as PA-457, although with a 2-fold higher efficacy for EP-47, compared to PA-457 and ST-327. However, the high cytotoxicity of EP-48, the beta-alanine-conjugated DSB, and of EP-62, the lysine-conjugated DSB with free lysine ε-NH2 group, made these compounds useless for further antiviral applications. Our EM observations showed that ST-327 and EP-47, as well as previously shown for PA-457 [12], blocked the virus assembly machinery in two cellular compartments, (i) at the plasma membrane when using budding-competent, N-myristoylated Gag precursor, and (ii) within the cytoplasm, when using budding-defective, non-N-myristoylated Gag precursor. This blockage occurred in a dose-dependent manner, with no detectable qualitative alterations of the VLP or core-like morphology [12].

The ethylene diamine-conjugated DSB (EP-39) was also found to inhibit the assembly of HIV-1 VLP, but in a manner which differed from that of the three other compounds PA-457, ST-327 and EP-47. EP-39 altered the viral assembly pathway in two manners. (i) It provoked the cytoplasmic assembly of an aberrant type of Gag particles never observed so far, morula-like particles of ca. 100-nm in diameter different in size and shape from the core-like particles formed of non-N-myristoylated Gag polyprotein [25]-[27], [32], [33], [53]. (ii) EP-39 did not inhibit the addressing to the cell surface of these morulae, but blocked their membrane wrapping, resulting in the release into the extracellular medium of uncoated Gag morula-like particles, recovered in the pelletable fraction in association with LucVpr. This explained the occurrence of a peak of luciferase activity co-sedimenting with Gag, and the apparent residual VLP production observed in the presence of EP-39, using our luciderase assays. *Bona fide* membrane-enveloped VLP and nonenveloped morula-like assemblies could easily be discriminated by their densities in isopycnic ultracentrifugation.

Ultrastructural analysis of the morula-like particles showed that they were composed of nanoparticular subunits of ca. 20-nm in diameter. The volume of these 20 nm-nanoparticles was theoretically sufficient to harbor 12 to 16 copies of Pr55Gag, arranged as a dodecahedron, decahedron, octahedron, or another type of solid of Platon. Biological nanoparticles composed of viral capsid components or subdomains thereof have already been described and characterized. E.g., twelve penton capsomeres of human adenovirus serotype 3 can form a symmetrical structure called penton dodecahedrons [72]–[74]. Likewise, dodecamers of P domain dimers of the protrusion (P) domain of the Norovirus capsid protein could assemble into octahedral structures of 20 nm in diameter, called Norovirus P nanoparticles. Each P nanoparticle of 24 subunits accommodated for a total protein mass of 840 kDa [75]. Based on this calculation, we estimated the Pr55Gag content of one nanoparticle of 19.4-nm in diameter to range from 12 (12×55 = 660 kDa) to 16 copies of Pr55Gag (16×55 = 880 kDa), arranged as a dodecahedron, decahedron, octahedron, or any other solid of Platon (**Fig. 10** **d**).

It was impossible to determine the exact number of nanoparticles per morula-like particles of Gag assembled in the presence of EP-39. However, there was no indication of an internal cavity within the morula, and a maximum of 20 individual beads could be simultaneously discerned on section planes of 100 nm-morulae, implying that the 60 nm-thick ultrathin section had passed through their equatorial plane. If we consider that one morula could be inscribed in a cube of 100 nm in edge, and since each edge would accomodate 5 beads each, each morula would theoretically contain 125 beads or nanoparticles of 20-nm (5×5×5 = 125), with a total number of Pr55Gag copies ranging between 1,500 (125×12) and 2,000 (125×16). Alternatively, if the morula adopted the structure of an icosahedron of roughly 100 nm in diameter and constituted of 180 nanoparticles, the total number of Pr55Gag copies would range between 2,160 (180×12) and 2,880 (180×16).

Of note, when administered to mammalian cells, EP39 also provoked aberrant assembly of HIV-1 particles: virions lacked conical cores, and instead contained spherical, acentric cores, and an additional electrondense layer beneath the viral envelope. However, no 100-nm morula-like or 20-nm nanoparticles were detected [55]. The structural differences with our recombinant Pr55Gag-expressing cells confirmed that viral proteins other than the Gag precursor, as well as the viral genome, contributed to virion formation [15], [76], and could partially compensate for the EP-39-induced Gag defect.

HIV-1 GagA364V is the prototype of a series of PA-457-resistant Gag mutants showing a normal or subnormal pattern of PR-mediated Gag processing in the presence of inhibitory doses of PA-457 [6], [11], [77], [78]. Based on the resistance to PA-457 conferred by A364V and other mutants of similar phenotype, the alanine residue at position 364 in the Pr55Gag sequence (position 1 of the SP1 domain) and its flanking regions in the CA and SP1 domains have been assumed to represent the main target of PA-457 [5], [6], [10], [11], [77], [78]. We therefore substituted the alanine residue for valine at codon-364 in the *gag* sequence of the baculoviral clone AcMNPV-Pr55Gag, in order to analyze the sensitivity of the GagA364V mutant to EP-39, in terms of assembly and budding of VLP from GagA364V-expressing cells. Unfortunately, we found that the GagA364V mutant was unstable in Sf9 cells, due to a premature cleavage near the CA-SP1 junction by cellular proteases, resulting in a major 41 kDa product (data not shown). This unstability prevented any study on the possible influence of the Ala-to-Val mutation at position 1 of the SP1 domain on the EP-39-mediated inhibition of VLP assembly.

Our preliminary NMR analysis indicated that the amino acid residues engaged in the interaction of Gag with EP-39 mapped to the SP1 domain (to be published). The EP-39 assembly inhibitor therefore provided some clues to the understanding of Gag interaction and oligomerization, and the possible mode(s) of Gag assembly at the polyprotein level, at steps preceding the formation of Gag immature particles [79]. Two hypotheses could be formulated to explain the pattern of Gag assembly in the presence of EP-39. (i) The 20 nm-nanoparticles might represent transient and/or labile Gag assembly intermediates which were blocked in their particular oligomeric status by EP-39. (ii) In a previous work, we showed that a Gag-derived polypeptide containing the SP1 domain could both dimerize and trimerize : contacts occurring at the hydrophobic surface of the region of CA-SP1 junction dimers would generate dimers, whereas trimers would result from contacts made at its hydrophilic surface [80]. EP-39 might block one or the other type of SP1-mediated inter-Gag contacts and favor one type of oligomerization process, or stabilize one particular form of Gag oligomers, e.g. the trimers. Isolation of 20 nm-nanoparticles induced by EP-39 is now in progress to determine their ultrastructure and type of symmetry using cryoelectron microscopy and tomography.

In conclusion, our study confirmed the necessity to develop sensitive and reliable assays to investigate HIV-1 virus assembly which were not simply based on CAp24 immunoassays. This would allow the quantitave evaluation of the efficacy of antivirals which target the final steps of HIV-1 productive cycle, assembly and budding. Our luciferase-Vpr packaging-based assay for the quantification of HIV-1 VLP production in insect cells led us to characterize a novel antiviral activity associated with EP-39, a derivative of betulinic acid corresponding to ethylene diamine-substituted PA-457. Our results demonstrated the importance of the side chains on carbon-28 of betulinic acid in the antiviral effect of DSB derivatives. Previous studies have shown that several carbon-28 substituants of betulinic acid have an antiviral effect at the post-entry step of HIV-1 infection, probably by interference with the viral-cell membrane fusion [49], [50], [81], [82]. Our data confirmed this membrane-targeting function, and suggested that betulinic acid would act as an anti-HIV prodrug with a multipolar activity, depending on its substituants. (i) Substituants on carbon-28, e.g. RPR-103611 [49], [50], [81], [82], would mainly interfere with membrane fusion, whereas (ii) substituants on carbon-3 (e.g. PA-457), and double substituants on both carbon-3 and carbon-28 (e.g. ST-327) would negatively interfere with virus maturation at low doses [5], [8], [10], [11], [55] and on immature particle assembly [12] and stability [13] at higher doses. (iii) The double substituant EP-39 showed a peculiar type of inhibitory effect, in that it altered not only the maturation and structure of the virus particles [55] but also the Gag precursor assembly, the membrane wrapping and budding process of immature Gag particles (this study). Our observation on the critical role of the side chain at position 28 in the anti-HIV-1 activity of betulinic acid derivatives had important implications in the design of future antivirals belonging to the betulinic acid family.

## Materials and Methods

### Chemical synthesis of betulinic acid (BA) derivatives

The leader compound 3-*O*-(3',3'-dimethylsuccinyl)-betulinic acid (DSB or PA-457; C_36_H_56_O_6_; MW = 584.8; DSB-_28_COOH) was synthesized from commercially available betulinic acid (BA; MW = 456.7; MP Biochemicals, France) as originally described [7], with some minor modifications reported in our previous study [12]. Glycine-conjugated DSB (DSB-_28_CO-Gly; ST-327; MW = 641.8), beta-alanine-conjugated DSB (DSB-_28_CO-βAla; EP-48; MW = 655.4), ethylene diamine-conjugated (DSB-_28_CO-EDA; EP-39; MW = 626.5), ε-N-*tert*-butoxycarbonyl-lysine-conjugated DSB (DSB-_28_CO-Lys-ε-NH-Boc; EP-47; MW = 812.5), and lysine-conjugated DSB with with free lysine ε-NH_2_ group (DSB-_28_CO-Lys; EP-62; MW = 712.5) were synthesized according to a published procedure [83], with some modifications. Detailed synthesis procedures will be published elsewhere [55], and vailable upon request. A schematic representation of these compounds is shown in **Fig. 5**.

### Cells and recombinant baculoviruses

***(i) Insect cells***. *Spodoptera frugiperda* Sf9 cells were maintained as monolayers, and infected with recombinant baculoviruses at a multiplicity of infection (MOI) ranging from 5 to 10 PFU/cell, as previously described [25], [26], [30], [32], [33]. ***(ii)*** ** ***Gag clones***. The HIV-1 *gag* gene, as well as the *luciferase-vpr* fusion gene, used in the present study were inserted into the genome of *Autographa californica* MultiCapsid NucleoPolyhedrosis Virus (AcMNPV) under the control of a chimeric AcMNPV-GmMNPV polyhedrin promoter, and the phenotypes of recombinant Gag proteins described in detail in previous studies [25], [26], [32], [33]. AcMNPV-Pr55Gag expressed the full-length wild type (WT) Gag polyprotein (Pr55Gag). The recombinant expressing the N-myristoylated version of the deletion mutant lacking the p6 carboxy-terminal domain, referred to as AcMNPV-GagΔp6(myr+) in previous studies [12], [28], [53] was simply referred to as AcMNPV-GagΔp6 for reason of acronym simplification. In AcMNPV-GagA364V mutant, the first residue of the SP1 domain, alanine, was mutated into valine, using the conventional PCR overlapping method, and the GagA364V mutation verified by DNA sequencing. ***(iii)*** ** ***Vpr***. The baculovirus clone expressing the oligohistidine-tagged Vpr protein (AcMNPV-Vpr) was obtained from Nathaniel Landau via Eric Cohen [84]. ***(iv)*** ** ***Luciferase-Vpr fusion construct (LucVpr)***. The plasmid carrying the *vpr* gene (LAI isolate) was obtained from Serge Benichou [47]. The firefly (*Photinus pyralis*) luciferase gene sequence was isolated by PCR from the pGL2 plasmid (control plasmid Cat # E1611; Promega). After deletion of its stop codon, a sequence coding for a 6-histidine tag and a GSGS linker was inserted at its 3'-end, and fused to the 5'-end of the *vpr* gene. The detail of this construct will be communicated upon request. The final fusion construct *luc(his)_6_-vpr* was inserted into AcMNPV to generate the AcMNPV-LucVpr recombinant. Of note, the reverse gene fusion *vpr-3*'*-luc(his)_6_* was also constructed, but the VprLuc fusion protein was found to be incapable of copackaging with Pr55Gag into VLP.

### Isolation of extracellular virus-like particles (VLP)

Sf9 cell culture supernatants were clarified by low-speed centrifugation, then VLP recovered using a two-step procedure comprising a sucrose-step gradient centrifugation [76], followed by an ultracentrifugation in linear D_2_O-sucrose gradient [12], [53]. (i) In the first step, VLP contained in the cell culture medium were pelleted through a sucrose cushion (20%, w:v, in TNE buffer; TNE: 100 mM NaCl, 10 mM Tris-HCl pH 7.4, 1 mM Na_2_EDTA) at 30 krpm for 1 h at 15°C in a Kontron TST55.5 rotor [12]. Pelleted VLP of step (i) were then gently resuspended in PBS (0.20–0.25 ml), and (ii) further analyzed by isopycnic ultracentrifugation in sucrose-D_2_O gradients [12], [53]. Linear gradients (10-ml total volume, 30–50%, w:v) were centrifuged for 18 h at 28 krpm in a Beckman SW41 rotor. The 50% sucrose solution was made in D_2_O buffered to pH 7.2 with NaOH, and the 30% sucrose solution was made in 10 mM Tris-HCl, pH 7.2, 150 mM NaCl, 5.7 mM Na_2_EDTA. Aliquots of 0.5 ml were collected from the top, and fractions analyzed for protein content by SDS-PAGE and immunoblotting, and by luciferase assay as described below.

### Gag assembly inhibition assays

Aliquots of Sf9 cells (10^6^) were infected with two recombinant baculoviruses at equal multiplicity of infection (MOI of 10 PFU/cell, each). One expressed the HIV-1 Gag precursor (AcMNPV-Pr55Gag or control, p6-deleted AcMNPV-GagΔp6), the other the control Vpr protein (AcMNPV-Vpr) or the LucVpr fusion (AcMNPV-LucVpr). At 24 h postinfection (pi), increasing quantities of PA-457, ST-327, EP-39 or EP-47 in DMSO were added to the infected cell cultures. To avoid any interference with a possible effect of DMSO, DMSO was kept constant in volume in the different samples. In standard experiments, stock solutions of PA-457, ST-327, EP-39 or EP-47 (10 mg/ml in DMSO) were diluted with DMSO to obtain a range of inhibitor concentrations from 0.5 to 20 µg per 2 µl-aliquot of DMSO, and each 2 µl-aliquot was added per 1 ml-volume of culture medium overlaying cell monolayers. Cells were harvested at 48 h pi, and extracellular VLP released in the culture medium were quantitated using luciferase assay. Membrane-enveloped VLP were pelleted and resuspended in lysis KDT buffer (KDT: 0.1 M potassium phosphate buffer, pH 7.8, 1 mM DTT, containing 0.2% Triton X100) for 30 min at 37°C with vortexing every 10 min. Luciferase activity associated with VLP was measured as previously described [85], using a Lumat LB-9501 luminometer (Berthold Technologies, Bad Wildbad, Germany). Of note, final concentrations of Triton X100 higher than 0.2% in samples were found to have a detrimental effect on the luciferase enzymatic activity. To compensate for eventual negative effects of drugs on LucVpr expression, the luciferase activity was measured in the corresponding cell lysates, obtained after lysis of the pelleted cells with KDT buffer. The results were expressed as relative light units (RLU) per µg protein. The values of the ratio of VLP-associated to intracellular luciferase activity were plotted versus the inhibitor concentration. The 50% inhibitory concentration (IC_50_) was defined as the compound concentration required to reduce this ratio by 50%, compared to the ratio obtained with untreated cells which was attributed the 100% value.

### Cytotoxicity assays

The cellular toxicity of the different compounds was evaluated using the MTT assays [86]. The 50% cytotoxic concentration (CC_50_) was defined as the concentration of the compound which reduced the cell viability by 50%, compared to that of untreated controls. The selectivity index (SI) was defined as the CC_50_ to IC_50_ ratio.

### Gel electrophoresis and quantitative assays of proteins

Polyacrylamide gel electrophoresis of SDS-denatured protein samples (SDS-PAGE), and immunoblotting analysis have been described in detail in previous studies [12], [25], [26], [53], [87]. Briefly, proteins were electrophoresed in SDS-denaturing, 10%- or 15%-polyacrylamide gel, along with prestained protein markers (PageRuler™ prestained protein ladder; Fermentas Inc., Hanover, MD, or Dual Color™ Standards, BioRad), and electrically transferred to nitrocellulose membrane (Hybond™-C-extra; GE Healthcare Bio-Sciences). Blots were blocked in 5% skimmed milk in Tris-buffered saline (TBS) containing 0.05% Tween-20 (TBS-T), rinsed in TBS-T, then successively incubated with primary rabbit or mouse anti-Gag antibodies, and relevant anti-IgG secondary antibodies, at working dilutions ranging from 1∶1,000 to 1∶10,000. Anti-HIV-1 Gag polyclonal antibody (laboratory-made; [53]) was raised in rabbit by injection of bacterially-expressed, GST-fused and affinity-purified C-truncated Gag protein consisting of full-length MA domain and the first seventy-eight residues of the CA domain (*Pst* I site; *gag*_Lai_ sequence). Mouse monoclonal antibody (mAb) anti-CAp24 (Epiclone #5001) and mAb anti-MAp17 (Epiclone #5003) were obtained from Cylex Inc. (Columbia, MD). Mouse monoclonal anti-oligo-histidine antibody was purchased from Quiagen S.A. (Courtaboeuf, France). Phosphatase-labeled anti-rabbit, or anti-mouse IgG conjugates were purchased from Sigma (St Louis, MO). For immunological quantification of VLP or VLP protein content, membrane-transferred protein were reacted with their specific primary antibody, then with ^125^I-labeled protein A (MP Biomedicals France, 67402 Illkirch; specific activity 30 µCi/µg) used at 20–30 µCi per 100 cm^2^ membrane, and exposed to radiographic films (Kodak BioMax HE film™, Sigma-Aldrich, 38297-St Quentin-Fallavier). Autoradiograms were scanned and quantitated by densitometric analysis, using the VersaDoc image analyzer and the Quantity One program (BioRad), or protein bands were excised from blots and radioactivity measured in a scintillation counter (Beckman LS-6500), as previously described [12], [53]. Alternatively, quantification of proteins in VLP was also performed using radiolabeling of proteins. Baculovirus-infected cells samples were labeled with ^35^S-amino acids (ICN Pharmaceuticals France, 91898-Orsay ; Tran^35^S-LABEL™; specific activity >1,000 Ci/mmol), added at 15 µCi/ml in methionine-free medium for 30 h at 18 h pi in the absence or presence of inhibitor in DMSO. VLP were recovered from the cell culture medium as described above, and radioactive proteins analysed by SDS-PAGE and autoradiography of dried gels.

### Electron microscopy and immuno-electron microscopy

Baculovirus-infected Sf9 cells were harvested at 48 h pi, pelleted, fixed with 2.5% glutaraldehyde in 0.1 M phosphate buffer, pH 7.5, post-fixed with osmium tetroxide (2% in H_2_O) and treated with 0.5% tannic acid solution in H_2_O. The specimens were dehydrated and embedded in Epon (Epon-812; Fulham, Latham, NY). Ultrathin sections were stained with 2.6% alkaline lead citrate and 0.5% uranyl acetate in 50% ethanol, and post-stained with 0.5% uranyl acetate solution in H_2_O [12], [28], [53]. Grids were examined under a Jeol JEM-1400 electron microscope, equiped with an ORIUS™ digitalized camera (Gatan France, 78113-Grandchamp). For statistical EM analyses, a minimum of 30 grid squares containing 10 to 20 cell sections each were examined for counting VLP budding at the cell surface, or budding in intracellular vesicular compartment.

For immuno-electron microscopy, specimens were fixed with 4% paraformaldehyde in 0.1 M phosphate buffer pH 7.3 for 4 h, post-fixed with 1% glutaradehyde in 0.1 M phosphate buffer pH 7.3 for 4 h, then rinsed overnight in 0.1 M phosphate buffer pH 7.3. After dehydration, specimens were included in LR White hydrophilic resin (Electron Microscopy Sciences, Hatfield, PA; EMS Catalog# 14380), and ultrathin sections deposited on nickel-coated grids. Grids were incubated with anti-Gag rabbit antibody (laboratory-made; [53]) at a dilution of 1∶50 in TBS for 1 h at room temperature (RT). After rinsing with TBS, the grids were post-incubated with 10-nm colloidal gold-tagged goat anti-rabbit IgG antibody (British Biocell International Ltd, Cardiff, UK; diluted to 1∶50 in TBS) for 30 min at RT. After rinsing with TBS, the specimens were post-stained with 1% uranyl acetate in H_2_0 for 1 min at RT, rinsed again with TBS, and examined under the electron microscope.

### Chemical cross-linking of intracellular Pr55Gag

Aliquots of recombinant baculovirus-infected Sf9 cells (5×10^5^ cells) untreated or treated with EP-39 at 10 µg/ml for 24 h at 24 h pi were centrifuged at low speed, resuspended in 200 µl of PBS containing the cross-linker bis(sulfosuccinimidyl)suberate (BS3; Pierce Biotechnology, Rockford, IL) at increasing molarities, ranging from 0 to 50 mM [88], and incubated in this buffer for 30 min at room temperature (RT). Cell samples were then centrifuged, resuspended and lysed in 200 µl 0.015 M NaCl, 0.5 mM phosphate buffer pH 7.3 containing BS3 at the same molarites as in the original mixtures, and further incubated for 30 min at RT. Cell lysates were mixed with 30 µl 6 times concentrated (6x) SDS-sample buffer without β-mercaptoethanol, heated to 100°C for 1 min, and Gag proteins analyzed by SDS-PAGE and immunoblotting. Western blots (Amersham Hybond™-ECL; GE-Healthcare) were incubated with polyclonal anti-Gag rabbit serum (laboratory-made; [53]) diluted to 1∶3,000 for 3 h at RT, followed by peroxidase-labeled anti-rabbit IgG (Sigma; dilution 1∶10,000) for 1 h at RT. Blots were then reacted with SuperSignal® West Pico chemiluminescence substrate (Pierce Biotechnology), and luminograms were visualized using the Fusion X7 imaging system with the Bio1D software (Vuilbert-Lourmat, Marne-la-Vallée, France).

## References

[1] Greene WC, Debyser Z, Ikeda Y, Freed EO, Stephens E (2008). Novel targets for HIV therapy.. Antiviral Res.

[2] Wainberg MA (2009). Perspectives on antiviral drug development.. Antiviral Res.

[3] Dorr CR, Yemets S, Kolomitsyna O, Krasutsky P, Mansky LM (2011). Triterpene derivatives that inhibit human immunodeficiency virus type 1 replication.. Bioorg Med Chem Lett.

[4] Kanamoto T, Kashiwada Y, Kanbara K, Gotoh K, Yoshimori M (2001). Anti-human immunodeficiency virus activity of YK-FH312 (a betulinic acid derivative), a novel compound blocking viral maturation.. Antimicrob Agents Chemother.

[5] Li F, Goila-Gaur R, Salzwedel K, Kilgore NR, Reddick M (2003). PA-457: a potent HIV inhibitor that disrupts core condensation by targeting a late step in Gag processing.. Proc Natl Acad Sci USA.

[6] Li F, Zoumplis D, Matallana C, Kilgore NR, Reddick M (2006). Determinants of activity of the HIV-1 maturation inhibitor PA-457.. Virology.

[7] Kashiwada Y, Hashimoto F, Cosentino LM, Chen CH, Garrett PE (1996). Betulinic acid and dihydrobetulinic acid derivatives as potent anti-HIV agents.. J Med Chem.

[8] Zhou J, Yuan X, Dismuke D, Forshey BM, Lundquist C (2004). Small-molecule inhibition of human immunodeficiency virus type 1 replication by specific targeting of the final step of virion maturation.. J Virol.

[9] Zhou X, Parent LJ, Wills JW, Resh MD (1994). Identification of a membrane-binding domain within the amino-terminal region of human immunodeficiency virus type 1 Gag protein which interacts with acidic phospholipids.. J Virol.

[10] Aiken C, Chen CH (2005). Betulinic acid derivatives as HIV-1 antivirals.. Trends Mol Med.

[11] Zhou J, Chen CH, Aiken C (2004). The sequence of the CA-SP1 junction accounts for the differential sensitivity of HIV-1 and SIV to the small molecule maturation inhibitor 3-O-{3',3'-dimethylsuccinyl}-betulinic acid.. Retrovirology.

[12] DaFonseca S, Blommaert A, Coric P, Hong SS, Bouaziz S (2007). The 3-O-(3',3'-dimethylsuccinyl) derivative of betulinic acid (DSB) inhibits the assembly of virus-like particles in HIV-1 Gag precursor-expressing cells.. Antiviral Ther.

[13] Keller PW, Adamson CS, Heymann JB, Freed EO, Steven AC (2011). HIV-1 maturation inhibitor bevirimat stabilizes the immature Gag lattice.. J Virol.

[14] Adamson CS, Freed EO (2010). Novel approaches to inhibiting HIV-1 replication.. Antiviral Res.

[15] Muriaux D, Darlix J-L, Cimarelli A (2004). Targeting the assembly of the human immunodeficiency virus type 1.. Current Pharmaceutical Design.

[16] Shvadchak V, Sanglier S, Rocle S, Villa P, Haiech J (2009). Identification by high throughput screening of small compounds inhibiting the nucleic acid destabilization activity of the HIV-1 nucleocapsid protein.. Biochimie.

[17] Tang C, Loeliger E, Kinde I, Kyere S, Mayo K (2003). Antiviral inhibition of the HIV-1 capsid protein.. J Mol Biol.

[18] Sticht J, Humbert M, Findlow S, Bodem J, Muller B (2005). A peptide inhibitor of HIV-1 assembly in vitro.. Nat Struct Mol Biol.

[19] Ternois F, Sticht J, Duquerroy S, Kräusslich H-G (2005). The HIV-1 capsid potein C-terminal domain in complex with a virus assembly inhibitor.. Nat Struct Mol Biol.

[20] Klikova M, Rhee SS, Hunter E, Ruml T (1995). Efficient in vivo and in vitro assembly of retroviral capsids from Gag precursor proteins expressed in bacteria.. J Virol.

[21] Jacobs E, Gheysen D, Thines D, Francotte M, de Wilde M (1989). The HIV-1 Gag precursor Pr55gag synthesized in yeast is myristoylated and targeted to the plasma membrane.. Gene.

[22] Sakuragi S, Sakuragi J, Morikawa Y, Shioda T (2006). Development of a rapid and convenient method for the quantification of HIV-1 budding.. Microbes Infect.

[23] Sakalian M, McMurtrey CP, Deeg FJ, Maloy CW, Li F (2006). 3-O-(3',3'-dimethysuccinyl) betulinic acid inhibits maturation of the human immunodeficiency virus type 1 Gag precursor assembled in vitro.. J Virol.

[24] Wilk T, Gross I, Gowen BE, Rutten T, de Haas F (2001). Organization of immature human immunodeficiency virus type 1.. J Virol.

[25] Carrière C, Gay B, Chazal N, Morin N, Boulanger P (1995). Sequence requirement for encapsidation of deletion mutants and chimeras of human immunodeficiency virus type 1 Gag precursor into retrovirus-like particles.. J Virol.

[26] Chazal N, Carrière C, Gay B, Boulanger P (1994). Phenotypic characterization of insertion mutants of the human immunodeficiency virus type 1 Gag precursor expressed in recombinant baculovirus-infected cells.. J Virol.

[27] Chazal N, Gay B, Carrière C, Tournier J, Boulanger P (1995). Human immunodeficiency virus type 1 MAp17 deletion mutants expressed in baculovirus-infected cells: *cis* and *trans* effects on the Gag precursor assembly pathway.. J Virol.

[28] Gay B, Tournier J, Chazal N, Carrière C, Boulanger P (1998). Morphopoietic determinants of HIV-1 GAG particles assembled in baculovirus-infected cells.. Virology.

[29] Gheysen D, Jacobs E, de Foresta F, Thiriart C, Francotte M (1989). Assembly and release of HIV-1 precursor Pr55gag virus-like particles from recombinant baculovirus-infected insect cells.. Cell.

[30] Hong SS, Boulanger P (1993). Self-assembly-defective dominant mutants of HIV-1 Gag phenotypically expressed in baculovirus-infected cells.. J Virol.

[31] Royer M, Bardy M, Gay B, Tournier J, Boulanger P (1997). Proteolytic activity in vivo and encapsidation of recombinant HIV-1 proteinase expressed in baculovirus-infected cells.. J Gen Virol.

[32] Royer M, Cerutti M, Gay B, Hong SS, Devauchelle G (1991). Functional domains of HIV-1 *gag*-polyprotein expressed in baculovirus-infected cells.. Virology.

[33] Royer M, Hong SS, Gay B, Cerutti M, Boulanger P (1992). Expression and extracellular release of human immunodeficiency virus type 1 Gag precursors by recombinant baculovirus-infected cells.. J Virol.

[34] Cohen EA, Dehni G, Sodroski JG, Haseltine WA (1990). Human immunodeficiency virus vpr product is a virion-associated regulatory protein.. J Virol.

[35] Kobinger GP, Borsetti A, Nie Z, Mercier J, Daniel N (1998). Virion-targeted viral inactivation of human immunodeficiency virus type 1 by using Vpr fusion proteins.. J Virol.

[36] Accola MA, Höglund S, Göttlinger HG (1998). A putative alpha-helical structure which overlaps the capsid-p2 boundary in the human immunodeficiency virus type 1 Gag precursor is crucial for viral particle assembly.. J Virol.

[37] Bachand F, Yao XJ, Hrimech M, Rougeau N, Cohen EA (1999). Incorporation of Vpr into human immunodeficiency virus type 1 requires a direct interaction with the p6 domain of the p55 gag precursor.. J Biol Chem.

[38] Jenkins Y, Pornillos O, Rich RL, Myszka DG, Sundquist WI (2001). Biochemical analyses of the interactions between human immunodeficiency virus type 1 Vpr and p6(Gag).. J Virol.

[39] Müller B, Tessmer U, Schubert U, Kräusslich H-G (2000). Human immunodeficiency virus type 1 Vpr protein is incorporated into the virion in significantly smaller amounts than Gag and is phosphorylated in infected cells.. J Virol.

[40] Mahalingam S, Khan SA, Murali R, Jabbar MA, Monken CE (1995). Mutagenesis of the putative alpha-helical domain of the Vpr protein of human immunodeficiency virus type 1: effect on stability and virion incorporation.. Proc Natl Acad Sci USA.

[41] Morellet N, Bouaziz S, Petitjean P, Roques BP (2003). NMR structure of the HIV-1 regulatory protein VPR.. J Mol Biol.

[42] Singh SP, Tomkowicz B, Lai D, Cartas M, Mahalingam S (2000). Functional role of residues corresponding to helical domain II (amino acids 35 to 46) of human immunodeficiency virus type 1 Vpr.. J Virol.

[43] Yao XJ, Subbramanian RA, Rougeau N, Boisvert F, Bergeron D (1995). Mutagenic analysis of human immunodeficiency virus type 1 Vpr: role of a predicted N-terminal alpha-helical structure in Vpr nuclear localization and virion incorporation.. J Virol.

[44] Accola MA, Bukovsky AA, Jones MS, Göttlinger HG (1999). A conserved dileucine-containing motif in p6(gag) governs the particle association of Vpx and Vpr of simian immunodeficiency viruses SIV(mac) and SIV(agm).. J Virol.

[45] Lu YL, Bennett RP, Wills JW, Gorelick R, Ratner L (1995). A leucine triplet repeat sequence (LXX)4 in p6 gag is important for Vpr incorporation into human immunodeficiency virus type 1 particles.. J Virol.

[46] Lu YL, Spearman P, Ratner L (1993). Human immunodeficiency virus type 1 viral protein R localization in infected cells and virions.. J Virol.

[47] Selig L, Pages J-C, Tanchou V, Prévéral S, Berlioz-Torrent C (1999). Interaction with the p6 domain of the Gag precursor mediates incorporation into virions of Vpr and Vpx proteins from primate lentiviruses.. J Virol.

[48] Votteler J, Studtrucker N, Sörgel S, Münch J, Rücker E (2007). Proline 35 of human immunodeficiency virus type 1 (HIV-1) Vpr regulates the integrity of the N-terminal helix and the incorporation of Vpr into virus particles and supports the replication of R5-tropic HIV-1 in human lymphoid tissue ex vivo.. J Virol.

[49] Mayaux J-F, Bousseau A, Pauwels R, Huet T, Henin Y (1994). Triterpene derivatives that block entry of human immunodeficiency virus type 1 into cells.. Proc Natl Acad Sci USA.

[50] Soler F, Poujade C, Evers M, Carry J-C, Henin Y (1996). Betulinic acid derivatives: a new class of specific inhibitors of human immunodeficiency virus type 1 entry.. J Med Chem.

[51] Lavallée C, Yao XJ, Ladha A, Göttlinger H, Haseltine WA (1994). Requirement of the Pr55gag precursor for incorporation of the Vpr product into human immunodeficiency virus type 1 viral particles.. J Virol.

[52] Paxton W, Connor RI, Landau NR (1993). Incorporation of Vpr into human immunodeficiency virus type 1 virions: requirement for the p6 region of gag and mutational analysis.. J Virol.

[53] Huvent I, Hong SS, Fournier C, Gay B, Tournier J (1998). Interaction and co-encapsidation of HIV-1 Vif and Gag recombinant proteins.. J Gen Virol.

[54] DaFonseca S, Coric P, Gay B, Hong SS, Bouaziz S (2008). The inhibition of assembly of HIV-1 virus-like particles by 3-O-(3',3'-dimethylsuccinyl) betulinic acid (DSB) is counteracted by Vif and requires its Zinc-binding domain.. Virol J.

[55] Turcaud S, Chazal N, Coric P, Souquet F, Briand L (2011). Synthesis of new derivatives of Bevirimat, showing a higher activity against HIV-1 maturation.. J Med Chem, submitted.

[56] Adamson CS, Freed EO (2007). Human immunodeficiency virus type 1 assembly, release and maturation.. Adv Pharmacol.

[57] Adamson CS, Jones IM (2004). The molecular basis of HIV capsid assembly - five years of progress.. Rev Med Virol.

[58] Cimarelli A, Darlix J-L (2002). Assembling the human immunodeficiency virus type 1.. Cell Mol Life Sci.

[59] Swanson CM, Puffer BA, Ahmad KM, Doms RW, Malim MH (2004). Retroviral mRNA nuclear export elements regulate protein function and virion assembly.. EMBO J.

[60] Wang CT, Stegeman-Olsen J, Zhang Y, Barklis E (1994). Assembly of HIV GAG-B-galactosidase fusion proteins into virus particles.. Virology.

[61] Larson DR, Johnson MC, Webb WW, Vogt VM (2005). Visualization of retrovirus budding with correlated light and electron microscopy.. Proc Natl Acad Sci USA.

[62] Perrin-Tricaud C, Davoust J, Jones IM (1999). Tagging the human immunodeficiency virus gag protein with green fluorescent protein. Minimal evidence for colocalisation with actin.. Virology.

[63] Alfadhli A, Dhenub TC, Still A, Barklis E (2005). Analysis of human immunodeficiency virus type 1 Gag dimerization-induced assembly.. J Virol.

[64] Fritz JV, Dujardin D, Godet J, Didier P, De Mey J (2010). HIV-1 Vpr oligomerization and not that of Gag directs the interaction between Vpr and Gag.. J Virol.

[65] Le Rouzic E, Benichou S (2005). The Vpr protein from HIV-1: distinct roles along the viral life cycle.. Retrovirology.

[66] Aguiar RS, Lovsin N, Tanuri A, Peterlin BM (2008). Vpr.A3A chimera inhibits HIV replication.. J Biol Chem.

[67] Wu X, Liu H, Xiao H, Kim J, Seshaiah P (1995). Targeting foreign proteins to human immunodeficiency virus particles via fusion with Vpr and Vpx.. J Virol.

[68] Yao XJ, Kobinger G, Dandache S, Rougeau N, Cohen E (1999). HIV-1 Vpr-chloramphenicol acetyltransferase fusion proteins: sequence requirement for virion incorporation and analysis of antiviral effect.. Gene Ther.

[69] Cavrois M, De Noronha C, Greene WC (2002). A sensitive and specific enzyme-based assay detecting HIV-1 virion fusion in primary T lymphocytes.. Nat Biotechnol.

[70] Briggs JA, Johnson MC, Simon MN, Fuller SD, Vogt VM (2006). Cryo-electron microscopy reveals conserved and divergent features of gag packing in immature particles of Rous sarcoma virus and human immunodeficiency virus.. J Mol Biol.

[71] Briggs JA, Simon MN, Gross I, Kräusslich H-G, Fuller SD (2004). The stoichiometry of Gag protein in HIV-1.. Nat Struct Mol Biol.

[72] Fender P, Ruigrok RW, Gout E, Buffet S, Chroboczek J (1997). Adenovirus dodecahedron, a new vector for human gene transfer.. Nat Biotechnol.

[73] Fender P, Schoehn G, Foucaud-Gamen J, Gout E, Garcel A (2003). Adenovirus dodecahedron allows large multimeric protein transduction in human cells.. J Virol.

[74] Fender P, Schoehn G, Perron-Sierra F, Tucker GC, Lortat-Jacob H (2008). Adenovirus dodecahedron cell attachment and entry are mediated by heparan sulfate and integrins and vary along the cell cycle.. Virology.

[75] Tan M, Huang P, Xia M, Fang P-A, Zhong W (2011). Norovirus P particle, a novel platform for vaccine development and antibody production.. J Virol.

[76] Muriaux D, Mirro J, Harvin D, Rein A (2001). RNA is a structural element in retrovirus particles.. Proc Natl Acad Sci USA.

[77] Adamson CS, Ablan SD, Boeras I, Goila-Gaur R, Soheilian F (2006). In vitro resistance to the human immunodeficiency virus type 1 maturation inhibitor PA-457 (Bevirimat).. J Virol.

[78] Adamson CS, Waki K, Ablan SD, Salzwedel K, Freed EO (2009). Impact of human immunodeficiency virus type 1 resistance to protease inhibitors on evolution of resistance to the maturation inhibitor bevirimat (PA-457).. J Virol.

[79] de Marco A, Müller B, Glass B, Riches JD, Kräusslich H-G (2010). Structural analysis of HIV-1 maturation using cryo-electron tomography.. PLoS Pathog.

[80] Morellet N, Druillennec S, Lenoir C, Bouaziz S, Roques BP (2005). Helical structure determined by NMR of the HIV-1 (345-392)Gag sequence, surrounding p2: implications for particle assembly and RNA packaging.. Protein Sci.

[81] Labrosse B, Pleskoff O, Sol N, Jones C, Henin Y (1997). Resistance to a drug blocking human immunodeficiency virus type 1 entry (RPR103611) is conferred by mutations in gp41.. J Virol.

[82] Yuan X, Huang L, Ho P, Labranche C, Chen CH (2004). Conformation of gp120 determines the sensitivity of HIV-1 DH012 to the entry inhibitor IC9564.. Virology.

[83] Jeong HJ, Chai HB, Park SY, Kim DS (1999). Preparation of amino acid conjugates of betulinic acid with activity against human melanoma.. Bioorg Med Chem Lett.

[84] Yao XJ, Rougeau N, Duisit G, Lemay J, Cohen EA (2004). Analysis of HIV-1 Vpr determinants responsible for cell growth arrest in *Saccharomyces cerevisiae*.. Retrovirology.

[85] Hong SS, Karayan L, Tournier J, Curiel DT, Boulanger PA (1997). Adenovirus type 5 fiber knob binds to MHC class I alpha2 domain at the surface of human epithelial and B lymphoblastoid cells.. EMBO J.

[86] Mosmann T (1983). Rapid colorimetric assay for cellular growth and survival: Application to proliferation and cytotoxicity assays.. J Immunol Methods.

[87] Peytavi R, Hong SS, Gay B, Dupuy d'Angeac A, Selig L (1999). H-EED, the product of the human homolog of the murine *eed* gene, binds to the matrix protein of HIV-1.. J Biol Chem.

[88] Knoller S, Shpungin S, Pick E (1991). The membrane-associated component of the amphiphile-activated, cytosol-dependent superoxide-forming NADPH oxidase of macrophages is identical to cytochrome b559.. J Biol Chem.

